# Atrial Myxoma: A Case Presentation and Review

**DOI:** 10.4021/cr145w

**Published:** 2012-01-20

**Authors:** Ronny Cohen, Gagandeep Singh, Derrick Mena, Christine A. Garcia, Pablo Loarte, Brooks Mirrer

**Affiliations:** a NYU School of Medicine, USA; b Woodhull Medical Center, USA; c St. George’s University School of Medicine, USA

**Keywords:** Atrial myxoma

## Abstract

Myxomas are the most common primary cardiac tumors, most frequently found in the left atrium. We present a case of an atrial myxoma. An in-depth review of atrial myxoma is presented, examining the important clinical symptoms and diagnostic indicators. The treatment of atrial myxoma is then discussed, with an emphasis on current therapies. An extensive literature review has been performed to present a comprehensive review of the causes, pathophysiology of atrial myxoma.

## Introduction

Myxomas are the most common primary cardiac neoplasm. The prevalence of cardiac tumors at autopsy ranges from 0.001% to 0.3%, more than 50% of benign cardiac tumors are myxomas. In 7%, it has genetic origin and rises as a component of a heritable disorder with some clinical manifestations. Over 72% of primary cardiac tumors are benign. In adults, the majority of benign lesions are myxomas [1, 2].

The origin can be explained through different theories. Myxomas are currently thought to originate from entrapped entrapped embryonic foregut, and hence they are derived from multipotent mesenchymal cells capable of both neural and epithelial differentiation. Histologically, these tumors are composed of scattered cells within a mucopolysaccharide stroma. Myxomas produce vascular endothelial growth factor (VEGF), which probably contributes to the induction of angiogenesis and the early stages of tumor growth [2-4].

On a macroscopic level, typical myxomas are pedunculated and gelatinous in consistency; the surface may be smooth, villous, or friable. Tumors vary widely in size, ranging from 1 to 15 cm in diameter, and weighing between 15 and 180 g. About 35 percent of myxomas are friable or villous, and these tend to present with emboli. Larger tumors are more likely to have a smooth surface and to be associated with cardiovascular symptoms [5].

### Clinical manifestations

Commonly observed symptoms and signs are dyspnea, orthopnea, paroxysmal nocturnal dyspnea, pulmonary edema, cough, hemoptysis, edema, and fatigue. Symptoms may be worsened in certain body positions, due to motion of the tumor within the atrium. On physical examination, a characteristic "tumor plop" may be heard early in diastole [6, 7]. Constitutional symptoms (e.g., fever, weight loss) are seen in around 30 percent of patients. Laboratory abnormalities (e.g., anemia and elevations in the erythrocyte sedimentation rate, C-reactive protein, or globulin level) are present in 35 percent, usually those with systemic symptoms [7].

There are several mechanisms by which cardiac tumors may cause symptoms. The obstruction of the circulation through the heart or heart valves produce symptoms of heart failure. Atrial myxoma may interfere with heart valves causing regurgitation. The direct invasion of the myocardium may result in impaired contractility, arrhythmias, heart block, or pericardial effusion with or without tamponade. The invasion of the adjacent lung may cause pulmonary symptoms and may mimic bronchogenic carcinoma. Finally, left atrial tumors may release tumor fragments or thrombi into the systemic circulation, leading to embolization which is usually systemic but can be pulmonic. The most serious complications of such embolization are neurologic. The rate of growth is unknown, as myxomas are mostly managed with surgical resection and only very rarely are medically managed due to contraindications to surgery [5, 8].

## Case Report

A 69 year old Hispanic female with past medical history of hyperthyroidism (Graves' Disease), heart failure with ejection fraction 45%, was presented to the emergency department with intermittent chest pain and palpitations. Chest pain was substernal, dull aching type, 5/10 in intensity and non-radiating.

Vital signs were stable with a blood pressure of 126/70 mmHg, pulse rate of 135 bpm, respiratory rate of 16 breaths per minute and temperature of 36.7 °C. On physical exam, pupils were equal, reactive to light and accommodation and notable for arcus senilis. There was no JVD present. Lungs were clear to auscultation bilaterally with no wheezing. Cardiovascular exam was notable for a grade 3/6, holosystolic murmur at the apex and irregularly irregular rhythm. Abdomen was soft, non-tender, non-distended with normal bowel sounds in all four quadrants. Pulses were intact bilaterally in upper and lower extremities with no edema.

Laboratory findings were as follows: WBC 7.5 K/ul, Hb 12.1 g/dl, Hct 36.8 %, Platelets 212 K/ul. Electrolyte panel findings were as follows: Na 143 mmol/L, K 4.0 mmol/L, bicarbonate 20 mmol/L, chloride 113 mmol/L, BUN 21 mg/dl, Cr 0.8 mg/dl, Glucose: 96 mg/dl. Other labs included: TSH 1.86 MIU/ml, Digoxin level < 0.1 ng/ml.

**Figure 1:**
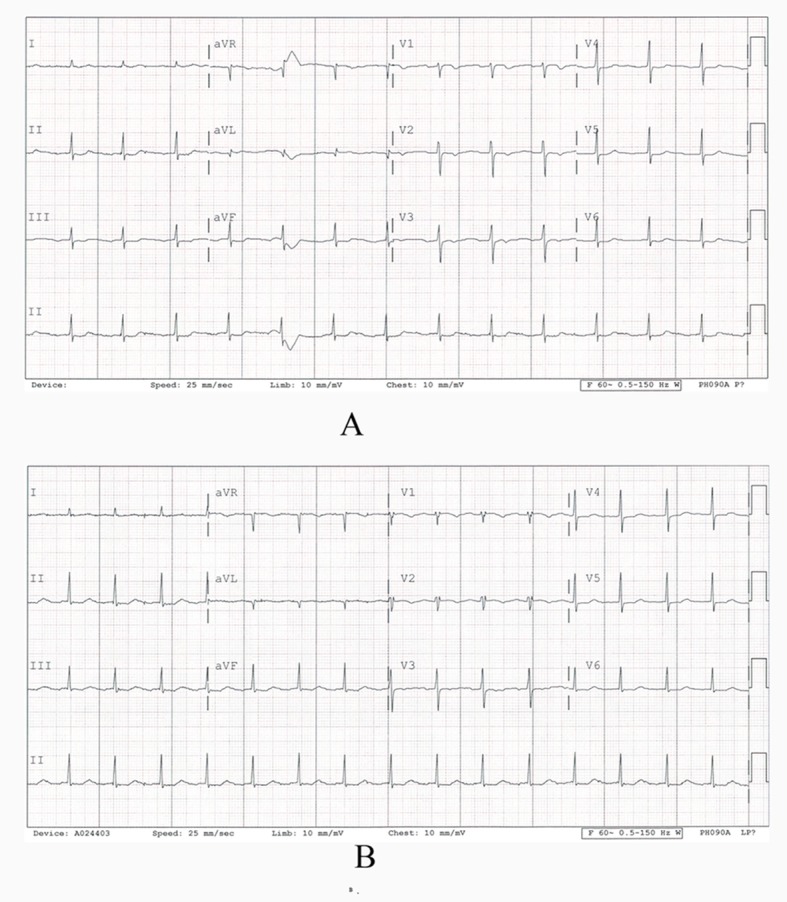
A: EKG showed atrial fibrillation with RVR at 135 bpm; B: EKG (post-op)

EKG showed atrial fibrillation with Rapid Ventricular Response at 135 bpm (Fig. 1).

**Figure 2:**
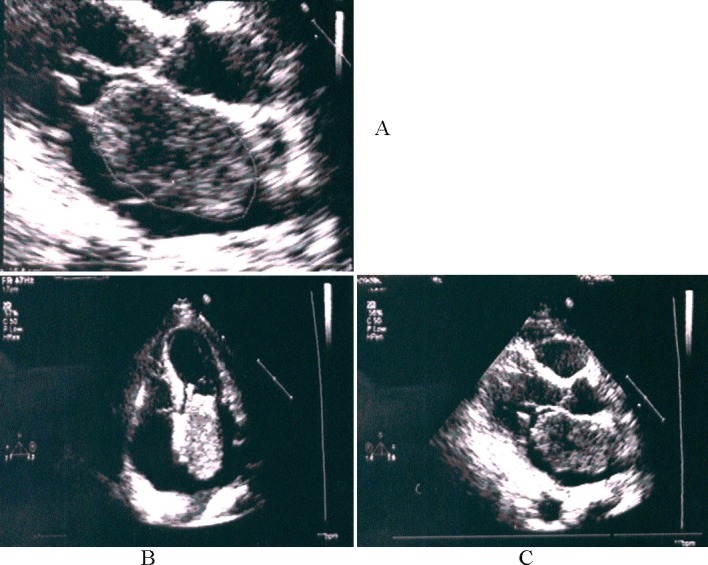
A: show a large mass 16 cm^2^ with attachment to the atrial septum; B: there is also a diastolic protrusion with obstruction towards left Long axis apical view during diastole; C: apical four chamber view during systole.

Transthoracic Echocardiogram (TTE) showed EF 45%, severe mitral regurgitation and a large mass in the left atrium, attached to interatrial septum, filling the whole chamber with slight protrusion into the anterior mitral valve leaflet and left ventricle during diastole (Fig. 2). It measured 16 cm^2^. Mitral valve diastolic velocity was increased to 2.5 m/s.

A diagnosis of left atrial myxoma was made and the case was referred to another hospital for surgical intervention. Patient was promptly transferred the next day and underwent resection of the left atrium myxoma and left atrial wall with pericardial reconstruction, mitral valve repair with # 24 Medtronic CG Future annuloplasty ring, left-sided modified MAZE RF atrial fibrillation ablation and amputation of left atrial appendage.

Post-operatively EKG showed Normal Sinus Rhythm at 80 bpm TTE showed no intracardiac mass and a normal left ventricle ejection fraction of 60%. Patient was started on anticoagulation and is being monitored in the outpatient clinic.

## Discussion

The patient presented with nonspecific chest pain symptoms. Common symptoms and signs of dyspnea, orthopnea, paroxysmal nocturnal dyspnea, pulmonary edema, cough, hemoptysis, edema, and fatigue lead to a wide differential diagnoses, making it critical for clinicians to be consider atrial mxyoma.

### Diagnostic evaluation

The goals of the initial evaluation are to ascertain whether or not a cardiac tumor is present, the location of the lesion within the heart and, to the extent possible, whether a tumor is benign or malignant. This information is vital in planning further evaluation and management. Echocardiography, cardiac MRI, and ultra-fast CT provide complementary information to address these questions.

#### Echocardiography

Echocardiography is widely available and provides a simple, noninvasive technique for the initial evaluation. Echocardiography images both the myocardium and the cardiac chambers can usually identify the presence of a mass. In addition, echocardiography may provide information about any obstruction to the circulation, as well as the likelihood that the tumor could be a source of emboli [9].

Although Transthoracic Echocardiography (TTE) is simple and usually can identify a tumor, Transesophageal Echocardiography (TEE) may be more informative. The superior diagnostic utility of TEE is due to the proximity of the esophagus to the heart, the lack of intervening lung and bone, and the ability to use high-frequency imaging transducers that afford superior spatial resolution. In our patient, the TTE was sufficient in confirming the diagnosis [9].

#### Cardiac MRI and Computed Tomography

Although both cardiac MRI and ultrafast CT provide noninvasive, high resolution images of the heart, MRI generally is preferred. In addition to furnishing detailed anatomic images, the T1- and T2-weighted sequences reflect the chemical microenvironment within a tumor, thereby offering clues as to the type of tumor that is present. However, CT scanning is still useful when MRI is not immediately available or is contraindicated [10, 11].

#### PET scan

Positron Emission Tomography (PET) has been useful in identifying cardiac involvement in patients with metastatic tumors, atrial myxoma or lipomatous septal hypertrophy [12].

#### Transvenous biopsy

Limited data is available on the risks and benefits of transvenous biopsy of suspected cardiac tumors. Because myxomas may embolize, transvenous biopsy is not generally warranted if the appearance is typical on noninvasive imaging. Biopsy is considered reasonable for other cardiac tumors if potential benefits are deemed sufficient to outweigh potential risks [13].

### Treatment and prognosis

Once a presumptive diagnosis of myxoma has been made on imaging studies, prompt resection is required because of the risk of embolization or cardiovascular complications, including sudden death. The results of surgical resection are generally very good, with most series reporting an operative mortality rate under 5 percent [11]. Cardiac transplantation has been reported for other tumors and might be considered for multiple, recurrent atrial myxomas [14].

Postoperative recovery is generally rapid. However, atrial arrhythmias or atrioventricular conduction abnormalities were present postoperatively in 26 percent of patients in one series [5]. In addition, patients are at risk for recurrence of the myxoma or the development of additional lesions. In one large series, 5 percent developed recurrent myxoma, suggesting the need for careful follow-up. Development of a second primary myxoma may be more common in patients with a family history of myxoma [15, 16].

## References

[1] Yu K, Liu Y, Wang H, Hu S, Long C (2007). Epidemiological and pathological characteristics of cardiac tumors: a clinical study of 242 cases. Interact Cardiovasc Thorac Surg.

[2] Amano J, Kono T, Wada Y, Zhang T, Koide N, Fujimori M, Ito K (2003). Cardiac myxoma: its origin and tumor characteristics. Ann Thorac Cardiovasc Surg.

[3] Mallick SR, Das P, Shukla B, Kothari S, Devagourou V, Ray R (2010). Right atrial myxoma with glandular differentiation: A rare entity in pediatric age group. Ann Pediatr Cardiol.

[4] Lloreta J, Juanpere N, Riverola A Cardiac myxoma with glandular differentiation: an immunohistochemical and ultrastructural Study. Ultrastructural Pathology.

[5] Pinede L, Duhaut P, Loire R (2001). Clinical presentation of left atrial cardiac myxoma. A series of 112 consecutive cases. Medicine (Baltimore).

[6] Kolluru A, Desai D, Cohen GI (2011). The etiology of atrial myxoma tumor plop. J Am Coll Cardiol.

[7] Aggarwal SK, Barik R, Sarma TC, Iyer VR, Sai V, Mishra J, Voleti CD (2007). Clinical presentation and investigation findings in cardiac myxomas: new insights from the developing world. Am Heart J.

[8] Burke AP, Gomez-Roman JJ, Loire R (2004). World Health Organization: Tumours of the lung, pleura, thymus and heart.

[9] Yoo M, Graybeal DF (2008). An echocardiographic-confirmed case of atrial myxoma causing cerebral embolic ischemic stroke: a case report. Cases J.

[10] Schroeder S, Achenbach S, Bengel F, Burgstahler C, Cademartiri F, de Feyter P, George R (2008). Cardiac computed tomography: indications, applications, limitations, and training requirements: report of a Writing Group deployed by the Working Group Nuclear Cardiology and Cardiac CT of the European Society of Cardiology and the European Council of Nuclear Cardiology. Eur Heart J.

[11] Rahmanian PB, Castillo JG, Sanz J, Adams DH, Filsoufi F (2007). Cardiac myxoma: preoperative diagnosis using a multimodal imaging approach and surgical outcome in a large contemporary series. Interact Cardiovasc Thorac Surg.

[12] Johnson TR, Becker CR, Wintersperger BJ, Herzog P, Lenhard MS, Reiser MF (2005). Images in cardiovascular medicine. Detection of cardiac metastasis by positron-emission tomography-computed tomography. Circulation.

[13] Patil HR, Singh D, Hajdu M (2010). Cardiac sarcoma presenting as heart failure and diagnosed as recurrent myxoma by echocardiogram. Eur J Echocardiogr.

[14] Reardon MJ, Malaisrie SC, Walkes JC, Vaporciyan AA, Rice DC, Smythe WR, DeFelice CA (2006). Cardiac autotransplantation for primary cardiac tumors. Ann Thorac Surg.

[15] Castells E, Ferran V, Octavio de Toledo MC, Calbet JM, Benito M, Fontanillas C, Granados J (1993). Cardiac myxomas: surgical treatment, long-term results and recurrence. J Cardiovasc Surg (Torino).

[16] Vohra HA, Vohra H, Patel RL (2002). Cardiac myxoma with three recurrences. J R Soc Med.

